# Transcriptional analysis of the expression and prognostic value of lipid droplet-localized proteins in hepatocellular carcinoma

**DOI:** 10.1186/s12885-023-10987-z

**Published:** 2023-07-18

**Authors:** Yize Zhang, Xue Liang, Qinghai Lian, Liwen Liu, Baoyu Zhang, Zihui Dong, Kunpeng Liu

**Affiliations:** 1 Precision Medicine Center, The First Affiliated Hospital of Zhengzhou University, Zhengzhou, China; 2 Gene Hospital of Henan Province, The First Affiliated Hospital of Zhengzhou University, Zhengzhou, China; 3 School of Life Science, Beijing University of Chinese Medicine, Beijing, China; 4 Cell-gene Therapy Translational Medicine Research Center, The Third Affiliated Hospital of Sun Yat-sen University, Guangzhou, China; 5 Department of Neurosurgery, The Third Affiliated Hospital of Sun Yat-sen University, Guangzhou, China; 6 School of Medicine, Guangxi University, Nanning, Guangxi China

**Keywords:** LDs-localized protein, NAFLD, HCC

## Abstract

The accumulation of lipid droplets (LDs) in hepatocytes is the main pathogenesis in nonalcoholic fatty liver disease (NAFLD), which is also the key risk factor for the progression of hepatocellular carcinoma (HCC). LDs behaviors are demonstrated to be associated with HCC advancement, and are tightly regulated by a subset protein localized on the surface of LDs. However, the role of LDs-localized protein in HCC has been rarely investigated. This study is focused on the transcriptional dynamic and prognostic value of LDs-localized protein in HCC. Firstly, we summarized the known LDs-localized proteins, which are demonstrated by immunofluorescence according to previous studies. Next, by the use of GEPIA/UALCAN/The Human Protein Atlas databases, we screened the transcriptional change in tumor and normal liver tissues, and found that 13 LDs-localized proteins may involve in the progression of HCC. Then we verified the transcriptional changes of 13 LDs-localized proteins by the use of HCC samples. Moreover, based on the assays of fatty liver of mice and human NAFLD liver samples, we found that the hepatic steatosis mainly contributed to the transcriptional change of selected LDs-localized proteins, indicating the involvement of these LDs-localized proteins in the negative role of NAFLD in HCC progression. Finally, we focused on the role of PLIN3 in HCC, and revealed that NAFLD status significantly promoted PLIN3 transcription in HCC tissue. Functional studies revealed that PLIN3 knockdown significantly limited the migration and chemosensitivity of hepatoma cells, suggesting the positive role of PLIN3 in HCC progression. Our study not only revealed the transcriptional change and prognostic value of lipid droplet-localized proteins in HCC, but also built the correlation between HCC and hepatic steatosis.

**Supplementary Information:**

The online version contains supplementary material available at 10.1186/s12885-023-10987-z.

## Introduction

Primary liver cancer, which is the seventh most frequently occurring type of cancer in the world, is the second most common reason of cancer mortality. Globally, hepatocellular carcinoma (HCC) is the dominant type of liver cancer, accounting for approximately 75% of the total liver cancer [1]. Because of the poor prognosis in all regions of the world, the incidence and mortality rates of HCC are roughly equivalent [2]. The occurrence and development of HCC can be induced by many factors, mainly including chronic hepatitis B and C (HBV, HCV), alcohol-related chronic liver disease, nonalcoholic fatty liver disease (NAFLD) and some other dietary factors [1]. Although HBV and HCV remain the most important risk factors for HCC at present, the prevalence of them should decline in the coming years due to vaccination and more effective treatment of both HBV and HCV carriers. Therefore, because of the increasing prevalence, NAFLD may gradually overtake the vital factor as the major cause of HCC all over the world [3, 4].

NAFLD usually begins with the accumulation of triglycerides in the liver, then further develops into nonalcoholic steatohepatitis (NASH), progresses to cirrhosis, and finally advances into HCC [5]. Previous studies mainly inclined to investigate the role of NAFLD in HCC tumorgenesis. Recently, the accompanying effects of NAFLD in HCC progression and immunotherapy have been reported increasingly [6]. Moreover, compared with other factors, NAFLD-associated HCC patients have shorter survival time, more advanced tumor stage, higher mortality and worse prognosis [7]. Therefore, it is of great clinical significance and social value to study the pathogenesis of NAFLD-associated HCC and further explore new drug targets for improving its diagnosis and treatment.

The most obvious feature of NAFLD is lipid metabolism dysfunction, which is closely related to a variety of liver diseases, including NAFLD-associated HCC [8, 9]. As important parts of lipid metabolism, LDs are existing as highly dynamic organelles, which are involved in the storage, transportation and metabolism of lipids [10]. LDs play important roles in the occurrence and development of NAFLD, and their continuous accumulation can further cause oxidative stress and inflammatory reaction in the liver, lead to sustained liver damage, and progress to more advanced states, such as NASH, fibrosis, and even HCC [11]. Notably, LDs accumulation in NAFLD-induced hepatoma or hepatoma occurrence accompanying NAFLD is common in the clinical practice, and it has been considered as main risk factors in HCC progression. Furthermore, it can also provide lipid substrate and energy sources for proliferation, migration and invasion of cancer cells [12]. In addition, some studies have shown that the accumulation of LDs can reduce drug-induced apoptosis and immunogenic cell death, leading to the chemoresistance of cancer cells [13, 14]. For now, although LDs have been considered to be important in the occurrence and development of HCC [15], the specific roles of LDs localized proteins in HCC are rarely investigated.

The function of LDs in cells is partly depends on LDs localized proteins, which can be divided into four categories: structural proteins, membrane transporters, enzymes and other proteins, such as histones, ribosomal proteins and signaling proteins [16]. The biological processes that LDs localized proteins participate in include lipid metabolism, redox metabolism, autophagy, gene transcription, ubiquitination, membrane transportation and immunity [17]. Some studies have shown that the abnormal expression of LDs localized proteins could lead to the aberrant accumulation and disorder function of LDs, which have been considered as new hallmarks of cancer [18, 19]. Although some studies have revealed that some LDs localized proteins has aberrant transcription and prognostic value in HCC, such as ACSL3 [20], HSD17B13 [21] or Rab10 [22], there are still few studies focusing on the panoramic value of LDs localized proteins in HCC progression, especially in NAFLD-accompanying HCC.

In this study, we firstly summarized the immunofluorescence-verified LDs localized proteins according to previous studies. Then we analyzed the transcriptional change and prognostic value of these LDs localized proteins, and found that 13 of them differentially expressed in HCC tissues, and had a high correlation with the prognosis of HCC patients. Moreover, we found the hepatic steatosis may contribute to the transcriptional change of the selected proteins. Furthermore, we took PLIN3 as the object to explore its function in the behavior of hepatoma cells. It has been found that PLIN3 could affect the invasion and chemosensitivity of hepatoma cells, indicating the participation of PLIN3 and other LDs localized proteins in HCC progression. Our studies not only provided evidences of the potential function of LDs localized proteins in hepatoma, but also revealed that these LDs localized proteins may be the key risk factors in the progression of NAFLD-accompanying hepatoma. Targeting to these LDs localized proteins may be a new therapeutic strategy for the precision medicine of NAFLD-accompanying HCC in the future.

## Materials and methods

### Literature search for selecting lipid droplet-associated factors

To determine the proteins present in lipid droplets, we conducted a comprehensive review of scholarly literature using the Google Scholar and PubMed databases. Our search utilized the keyword “lipid droplet localized protein” and was last updated in December 2022. Our selection criteria for protein inclusion extended beyond MS-identification and encompassed immunofluorescence evidence demonstrating co-localization of the protein and lipid droplets. Through careful analysis and interpretation of the articles retrieved, we were able to identify and select 38 proteins exhibiting immunofluorescence evidence of co-localization with lipid droplets.

### GEPIA (Gene expression profiling interactive analysis)

The gene expression profiling in HCC tumor and normal tissues and overall survival analysis were obtained from the GEPIA platform (http://gepia.cancer-pku.cn/), which is an online resource for gene expression analysis based on bulk tumor an normal samples based on TCGA and GTEx databases [23]. Our analysis included 369 liver cancer samples and 50 normal liver tissue samples. The mRNA expression profiles of selected LDs localized proteins were generated between liver cancer and normal tissues, and the statistical analysis was performed through the plug-in units of GEPIA. Furthermore, they were also analyzed for overall survival (OS) by using the Kaplan-Meier survival plots tool in the GEPIA platform.

### UALCAN

UALCAN (http://ualcan.path.uab.edu), an interactive online database based on RNA-seq and clinical data of 31 cancer types for in-depth analysis of TCGA database [24]. Our research used UALCAN to analyze the mRNA expression of the selected LDs localized proteins in different tumor grades and tumor stages of HCC patients. Differences in transcriptional expression were compared by Student’s t-test, and the statistically significant was represented by *p < 0.05, **p < 0.01, and ***p < 0.001.

### Survival analysis

An investigation was conducted on the prognostic value of 12 genes selected for HCC patients, utilizing the TCGA database. The TCGA database of liver hepatocellular carcinoma (LIHC) contains RNA-Seq data for 374 HCC patients and 50 normal tissues (https://portal.gdc.cancer.gov/) for gene expression and immune system infiltrates. We determined the correlations between mRNA levels of LD-associated proteins expression and the survival of HCC patients using the KM Plotter (http://www.kmplot.com) (accessed on 18 December 2021) databases. Additionally, a time-dependent receiver operating characteristic (ROC) analysis (including 1-, 3-, and 5-year survival) was conducted to evaluate selected gene sensitivity and specificity using the R package “survival ROC” based on the TAGA dataset.

### Patients and specimens

The liver tissue cohort (liver normal tissues, NAFL tissues, HCC tissues with or without NAFL) in this study was obtained from the patients who underwent surgical resection at the Third Affiliated Hospital of Sun Yat-sen University from January 2013 to December 2016. The patients neither had any history of other malignancies nor received any treatment when enrolled. All experimental procedures complied with the experimental management regulations of the State Council of the People’s Republic of China, and strictly follow China’s GCP, the “Declaration of Helsinki” and relevant Chinese laws and regulations. The usage of the clinical samples and methods in this study was carried out in accordance with the approved guidelines by the Third Affiliated Hospital of Sun Yat-Sen University, and written informed consent to participate in the study was obtained from all subjects. Informed consent forms were signed by all study participants. The protocol was approved by the Institutional Review Board of the Third Affiliated Hospital of Sun Yat-sen University. All of the collected tissues were used for RNA extraction and qRT-PCR, immunohistochemistry, and western blot, respectively.

### RNA extraction and qRT-PCR analysis

RNA from fresh frozen tissues was extracted by TRIzol™ reagent (Thermo Fisher, 15,596,026, USA). Reverse transcription was performed with Random Hexamer Primer and RevertAid First Strand cDNA Synthesis Kit (Thermo Fisher, k1622, USA) according to the manufacturer’s instruction, and the resulting cDNAs were subjected to qRT-PCR analysis with FastStart SYBR Green Master (Roche, 04673484001, Germany) in LightCycler 96 Real-Time PCR system (Roche, D10013, Germany). The amplification primers were purchased from Sangon Biotech, China. The expression levels were normalized to those of RPL13A. The amplification primers were as follow:

**Table:** 

Gene Name	Forward Primer (5’ to 3’)	Reverse Primer (5’ to 3’)
ACSL3	GCCGAGTGGATGATAGCTGC	ATGGCTGGACCTCCTAGAGTG
AIFM2	GTGAGCGGGTGAGCAATCT	CTTGATGCCGGTGCAGAGAA
CIDEB	CAGCGACCTTTCCGTGTCT	GGGTCTCCAATGCTTTGGCT
G0S2	CCTGATGGAGACTGTGTGCAG	CCTGCTGCTTGCCTTTCTC
HILPDA	AAGCATGTGTTGAACCTCTACC	TGTGTTGGCTAGTTGGCTTCT
HSD17B7	ATCTGGACATCATCTCGCAGT	AAGAGCTGTAGGGTTCCTTGC
HSD17B13	CTCATCCCATATTGTTCCAGCAA	AGGCCATAATCTTGTGCTTGG
LPCAT1	TTACCTTCAAACCTGGTGCATT	CGTGAGCCACAGGATTTCC
LSS	ACATTGAGGATAAGTCCACCGT	TCGTACCAGGTCAGGATCGTC
PLIN1	TGTGCAATGCCTATGAGAAGG	AGGGCGGGGATCTTTTCCT
PLIN2	ATGGCATCCGTTGCAGTTGAT	GGACATGAGGTCATACGTGGAG
PLIN3	GCCCAAGAGATGGTGTCTAGC	CCGGTCACTACGGACTTTGT
RAB10	CTGCTCCTGATCGGGGATTC	TGATGGTGTGAAATCGCTCCT
RPL13A	GCCATCGTGGCTAAACAGGTA	GTTGGTGTTCATCCGCTTGC

### Immunohistochemistry (IHC) and Immune scores

Representative immunohistochemistry of selected LDs localized proteins between liver cancer and normal liver tissues was obtained from The Human Protein Atlas (http://www.proteinatlas.org/).

PLIN3 IHC staining was performed as described before [25]. In brief, the liver sections were deparaffinized, hydrated, and incubated with 3% hydrogen peroxide solution to quench the endogenous peroxidase activity, followed by antigen retrieval in 10 mM citrate buffer (pH = 6.0) at 95℃ and blocked in 5% BSA (Solarbio, SW3015, China) at room temperature to prevent nonspecific binding. Subsequently, the sections were incubated with the primary rabbit polyclonal antibody anti-PLIN3 (20 µg/ml, Abcam, ab47638, UK) overnight at 4℃. The secondary antibody (HRP-conjugated goat anti-rabbit IgG, 1:500 dilution, Beyotime, A0208, China) was added at room temperature, and then the sections were developed with 3, 3’-diaminobenzidine (DAB) (Beyotime, P0203, Chine) for staining.

The immune scores were blindly calculated by different individuals using the staining intensity multiplied by the cell percentage (+) %*1 + (++) %*2 + (+++) %*3. + indicates a weak staining signal; ++ indicates a medium staining signal; +++, indicates a strong staining signal.

### Western blot analysis

Total protein of each sample (tissues or cell lysates) was obtained by using RIPA lysis buffer (Solarbio, R0010, China) and quantified by performing BCA protein assay kit (Solarbio, PC0020, China). After denaturation, an equal amount of protein from each sample was separated on SDS-PAGE gel and transferred to a methanol-activated PVDF membrane (Merk Millipore, ISEQ00010, USA) by electroblotting. The membrane was then blocked in 5% nonfat milk powder in 10 mM PBS buffer at 4℃ overnight. The blocked membrane was then incubated sequentially with the diluted primary antibodies PLIN3, (Abcam, ab47638, UK); c-PARP (Cell Signaling Technology, 5625, USA); c-Caspase 3 (Cell Signaling Technology, 9664, UK); β-Actin (Proteintech, 66009-1-Ig, China); β-tubulin, (Proteintech, 10094-1-AP, China) and HRP-conjugated second antibody (Beyotime, A0208, China). Finally, the immunoreactive protein bands were detected by BeyoECL Plus kit (Beyotime, P0018S, China) according to the manufacturer’s instructions. The original western blot bands were provided in the supplemental information.

### Cell culture and transfection

Human HCC cell lines, HepG2 and LM3 were obtained from the Cell Bank of the Chinese Academy of Science, Shanghai, China. They were cultured in DMEM medium (Procell, PM150210, China) containing 10% fetal bovine serum (ExCell, FSP500, China) and 100 U/ml penicillin/streptomycin (Hyclone, SV30010, USA) in a 5% CO_2_ humidified incubator at 37℃.

PLIN3 silencing was achieved by the transfection of a specific lentiviral vector containing a short hairpin RNA (shRNA) targeting PLIN3 (Hanbio Tech, China) combined with Lipofiter™ (Hanbio Tech, China). The selection for PLIN3 sh-RNA or negative control transfected cells were processed by using puromycin at 2 µg/ml. The target sequences for PLIN3 were exhibited as follows:

NC: 5’-TTCTCCGAACGTGTCACGTAA-3’;

PLIN3-sh1: 5’-ACCGTGTGGCCAGCATGCCTCTGAT-3’;

PLIN3-sh2: 5’-CCAAGGAGAGCTACCCGCACATCAA-3’.

### Cell proliferation, Migration and Invasion assays

Cell proliferation performed by Cell Counting Kit-8 (CCK-8) assay, 4 × 10^3^ HepG2 and LM3 negative control cells or PLIN3 knockdown cells were respectively cultured in 96-well plates and incubated with Cell Counting Kit-8 (Beyotime, C0038, China) for 2 h at 37℃. Cell viability was measured with the absorbance at the wavelength of OD450 nm on a spectrophotometer at 0 h, 24 and 48 h. The chemosensitivity of PLIN3 knockdown cells was tested by the treatment with sorafenib (Selleck, S7397, China), doxorubicin (Selleck, E2516, China), or cisplatin (Selleck, S1166, China) at 0 µM, 0.1 µM, 1 µM, 10 µM and 50 µM for 48 h, and the cell viability was measured as described before.

Cell migration and invasion assays were performed by using the transwell chambers with 8-µm pores (Corning, 3422, USA) coated without or with the Matrigel (BD, 356,234, USA) (1:40 diluted by DMEM medium) respectively. 3 × 10^4^ cells mixed in DMEM medium without FBS were plated into the upper sections, and DMEM medium with 10% FBS was loaded in the lower chambers. The chambers were then incubated for 24 h and after the fixation with paraformaldehyde for 20 min, the cells were stained with crystal violet for 15 min and then counted by using optical microscope.

### NASH model

Total 12 six-week old C57BL/6 mice were randomly divided into control and experimental groups (6 per group) treated with CSAA (Dyets, 518,754, USA) and CDAA (Dyets, 518,753, USA) feed respectively. After one week acclimatization, the experimental group was to be fed with the transitional feed for one week. During this transitional stage, the CSAA and CDSS feeds were mixed at 2:1 on the first three days, 1:1 on the fourth to fifth days, 1:2 on the sixth to seventh days, and CDAA feed was completely added after this period. At the 6th week after transitional feeding, the animals of each group were sampled. The pathological feature and steatosis of the mouse livers were confirmed by H&E staining and Oil red staining, respectively. Furthermore, mRNA expression of the selected LDs localized proteins was examined by the transcriptome.

### Statistical analysis

Data were analyzed by Student’s *t*-test using the GraphPad Prism 7 (San Diego, USA). The significance was set at *p < 0.05, **p < 0.01, and ***p < 0.001.

## Results

### The summarization of LDs localized proteins verified by immunofluorescence

Although many studies identified the LDs-associated proteins by using of mass spectrum, only few proteins have been demonstrated to localize on the surface of LDs, which is key to participate in the regulation of LDs’ dynamic. To identify the verified protein localized on lipid droplet, we performed a literature mining in the previous publications related to lipid droplet-associated proteins verified by immunofluorescence (last search date: December 2022) (Table 1). Next, we performed transcriptional analysis of these proteins which differentially expressed in HCC.

**Table 1:** LDs localized proteins verified by immunofluorescence

Proteins	PMID	Proteins	PMID	Proteins	PMID
ABHD5	26,350,461	HILPDA	33,465,519	PLIN2	25,338,003
ACSL3	22,357,706	HSD17B11	18,804,447	PLIN3	19,451,273
AIFM2	29,275,994	HSD17B7	29,275,994	PLIN5	21,885,430
AUP1	29,902,443	HSD17B13	32,973,038	PNPLA2	18,980,248
C18orf32	29,275,994	LDAH	24,357,060	PNPLA3	31,019,090
CAV1	15,111,495	LIPE	12,810,697	Rab1B	29,275,994
CIDEB	19,187,774	LPCAT1	21,498,505	Rab5A	29,275,994
ZFYVE1	31,293,035	LPCAT2	21,498,505	Rab7A	27,453,349
DGAT2	229,277,462	LSS	29,275,994	Rab10	28,028,537
DHRS3	21,659,514	METTL7A	18,477,614	Rab18	29,367,353
FAF2	23,297,223	METTL7B	18,477,614	RDH11	29,275,994
G0S2	20,197,052	PITPNB	29,275,994	SCCPDH	29,275,994
AGPAT6	23,415,954	PLIN1	23,481,402		

### Transcriptional level of lipid droplets localized genes in liver hepatocellular carcinoma

**Fig. 1:**
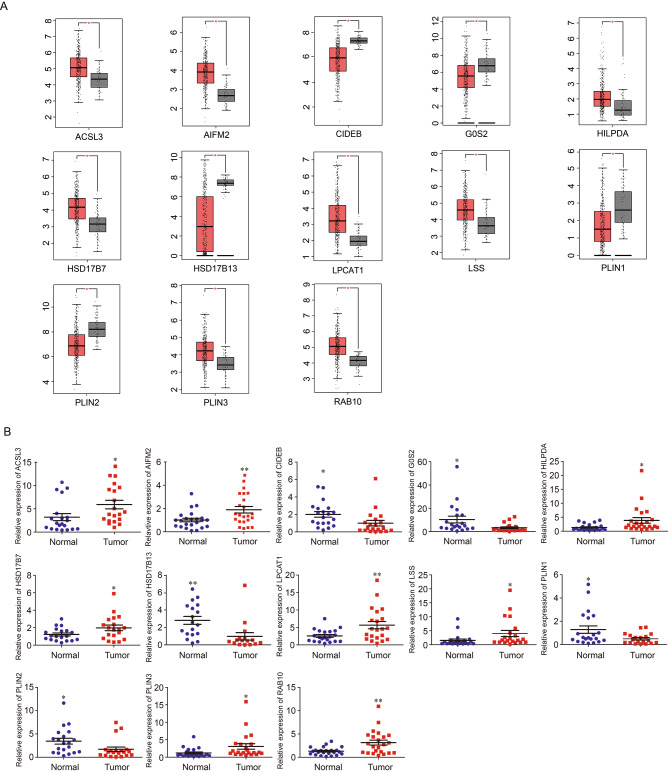
The mRNA expression levels of selected proteins differentially expressed in HCC by analysis of mRNA sequence data. (**A**) mRNA expression levels of 13 differentially expressed lipid droplets localized genes in liver hepatocellular carcinoma. Gene mRNA expression level was analyzed with the GEPIA platform. The 13 differentially expressed genes (*ACSL3*, *AIFM2*, *CIDEB*, *G0S2*, *HILPDA*, *HSD17B7*, *HSD17B13*, *LPCAT1*, *LSS*, *PLIN1*, *PLIN2*, *PLIN3*, *RAB10*) were depicted via bar plots between liver tumor (red column, n = 369) versus liver normal tissue (grey column, n = 50). (**B**) Relative mRNA levels of 13 differentially expressed lipid droplets localized genes in liver normal tissues (n = 23) versus tumor tissues (n = 23). The mRNA expression levels were determined by real-time RT-PCR. *p < 0.05, **p < 0.01

**Fig. 2:**
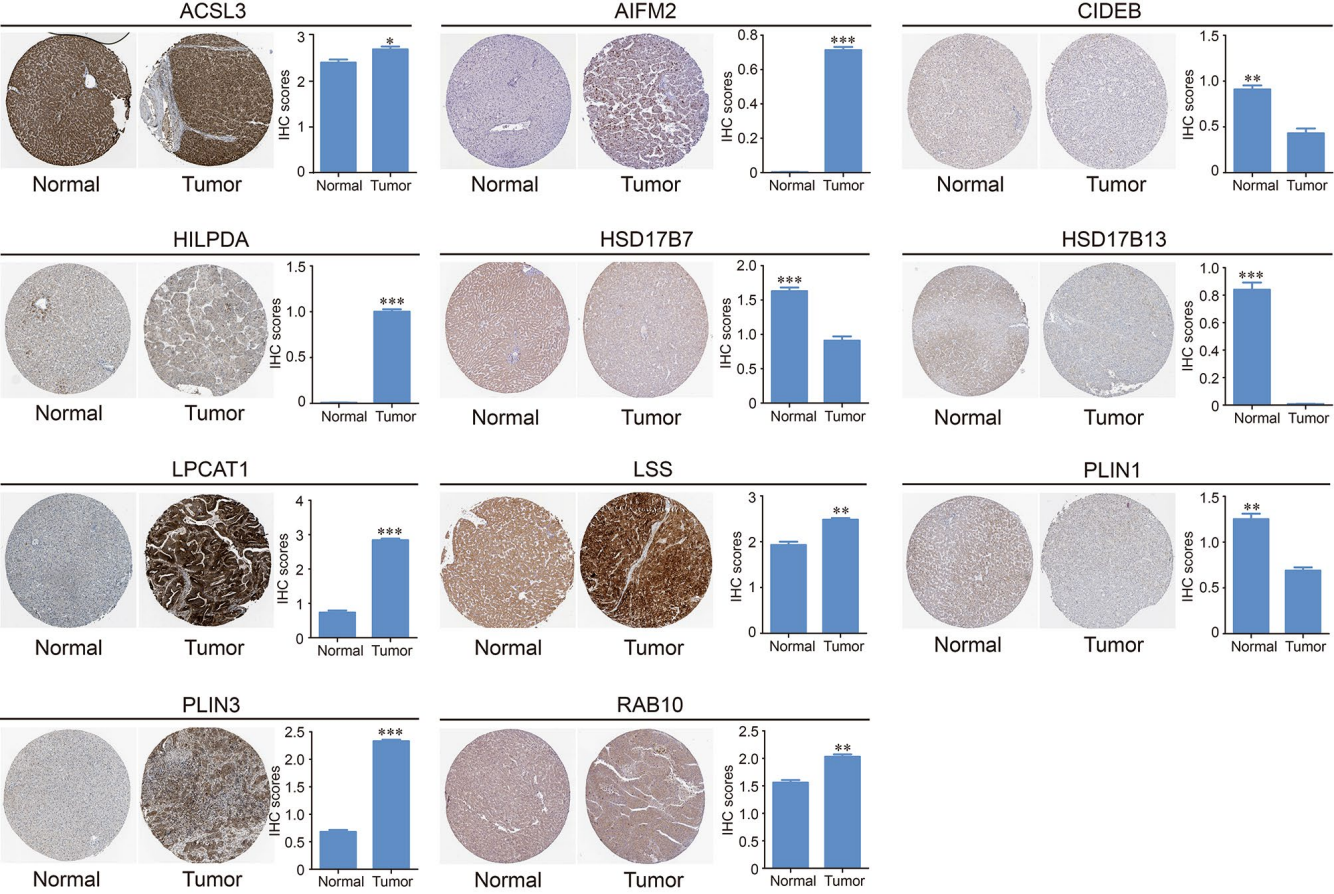
Representative immunohistochemistry of differentially expressed proteins localized on lipid droplets between liver normal tissues and liver tumor tissues in the Human Protein Atlas database

To identify the role of LDs localized proteins, we performed a transcriptional analysis of these proteins in the GEPIA database and selected 13 LDs localized proteins which were differentially expressed in liver normal and tumor tissues. Based on GEPIA platform, we further analyzed the transcriptional levels of selected lipid droplets localized genes in patients with HCC. Data from GEPIA showed that 8 genes (*ACSL3*, *AIFM2*, *HILPDA*, *HSD17B7*, *LPCAT1*, *LSS*, *PLIN3*, and *RAB10*) were up-regulated, and 5 genes (*CIDEB*, *G0S2*, *HSD17B13*, *PLIN1*, and *PLIN2*) were down-regulated in bulk LIHC tissues (n = 369) compared with the adjacent normal tissues (n = 50) (Fig. 1A). Furthermore, we applied q-PCR to validate the expression of these genes in hepatoma tissue of HCC patients collected by our group. The result indicated that the expression patterns of selected 13 lipid droplets localized genes were consistent with the data from GEPIA (Fig. 1B). We further analyzed the protein expression of the 13 selected lipid droplets localized genes on the Human Protein Atlas database. The representative immunohistochemistry pictures and the IHC scores were shown in Fig. 2. The IHC information of G0S2 and PLIN2 were not found in this database. Altogether, these data revealed that 13 proteins among the selected cohort of LDs localized proteins have transcriptional change in HCC, indicating that the selected proteins may have function in the progression of HCC, and may correlated with the prognosis of HCC patients.

### Relationship between transcriptional levels of lipid droplets localized genes and the clinicopathological parameters in HCC patients

**Fig. 3:**
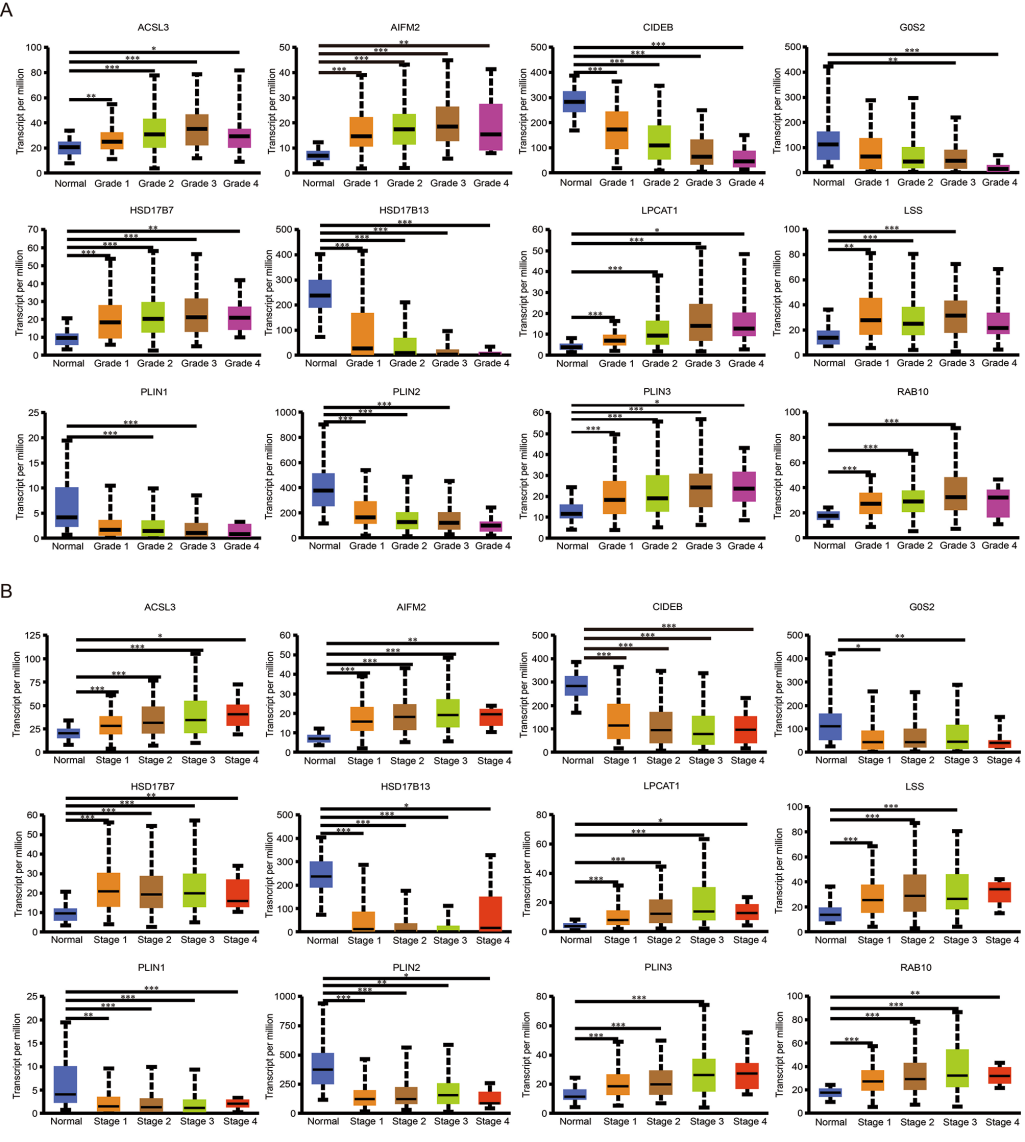
The transcriptional association between selected genes and HCC progression was analyzed in UALCAN. (**A**) Relationship between the mRNA expression of 12 differentially expressed lipid droplets localized genes and tumor grade of HCC patients. Normal: liver normal tissues (n = 50); Grade 1: well differentiated (low grade, n = 54); Grade 2: moderately differentiated (intermediate grade, n = 173); Grade 3: poorly differentiated (high grade, n = 118); Grade 4: undifferentiated (high grade, n = 12). (**B**) Relationship between the mRNA expression of 12 differentially expressed lipid droplets localized genes and individual cancer stage of HCC patients. Normal: n = 50; Stage 1: n = 168; Stage 2: n = 84; Stage 3: n = 82; Stage 4: n = 6. *p < 0.05, **p < 0.01, ***p < 0.001

According to UALCAN database, we analyzed the relationship between mRNA expression of selected lipid droplets localized genes and the clinicopathological parameters of HCC patients, including the tumor grade and individual cancer stage. The mRNA expression levels of selected lipid droplets genes were significantly correlated with the tumor grade of HCC patients. The highest mRNA levels of *ACSL3*, *AIFM2*, *HSD17B7*, *LPCAT1*, *LSS*, *PLIN3*, and *RAB10* were found in grade 3 tumors, while the lowest mRNA levels of *CIDEB*, *G0S2*, *HSD17B13*, *PLIN1* and *PLIN2* were found in grade 4 tumors (Fig. 3A). Similarly, the mRNA levels of selected localized genes were also related to individual cancer stage. *ACSL3*, *AIFM2*, *HSD17B7*, *LPCAT1*, *LSS*, *PLIN3* and *RAB10* were up-regulated with the progression of cancer stage of HCC patients, while *CIDEB*, *G0S2*, *HSD17B13*, *PLIN1* and *PLIN2* were down-regulated (Fig. 3B). Regretfully, we have not found the information of *HILPDA* in this database. In summary, these results indicated that the mRNA levels of selected lipid droplets localized genes were significantly associated with the clinicopathological parameters in HCC patients.

### The prognostic value of lipid droplets genes for OS in LIHC patients

**Fig. 4:**
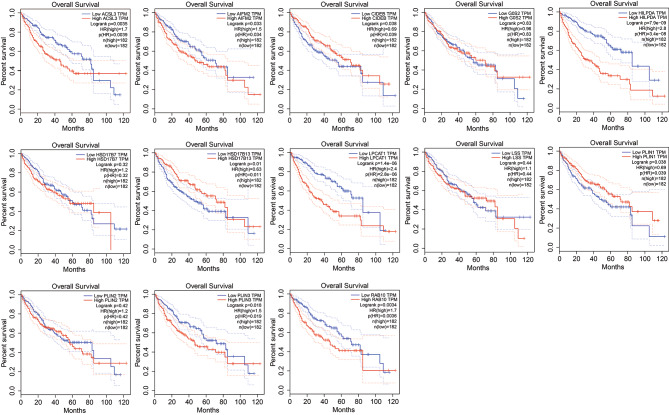
The OS prognostic values of 13 differentially expressed lipid droplets localized genes in LIHC were analyzed by GEPIA.

GEPIA database was used to analyzed the relationship between the expression of each selected lipid droplets gene and patients OS in LIHC. The results revealed that high expression of *ACSL3*, *AIFM2*, *HILPDA*, *LPCAT1*, *PLIN3* and *RAB10*, and low expression of *CIDEB*, *HSD17B13*, and *PLIN1* were significantly associated with poor OS of patients with LIHC. While the expression of *G0S2*, *HSD17B7*, *LSS* and *PLIN2* was not related to the OS of LIHC patients (Fig. 4).

A comprehensive investigation was conducted to explore the correlation between selected genes and the prognosis of HCC patients. The progression-free interval (PFI), disease-specific survival (DSS), and relapse-free survival (RFS) were analyzed for the identified proteins in HCC patients. Our findings suggest that the transcription of HILPDA, HSD17B13, LPCAT1, PLIN3, and RAB10 may be associated with the progression-free survival of HCC patients (Figure S2A). Similarly, DSS analysis showed that the transcription of HILPDA, HSD17B13, LPCAT1, and RAB10 may be associated with the disease-free survival of HCC patients (Figure S3A). Additionally, the transcription of CIDEB, HSD17B13, LPCAT1, and RAB10 may be associated with the relapse-free survival of HCC patients, as demonstrated by RFS analysis (Figure S4A). For determined the prognostic value of selected genes in HCC patients, we used the whole TCGA dataset of LIHC (374 HCC patients) for time-dependent ROC analysis, and found that the AUC scores of ACSL3, HILPDA, LPCAT1, PLIN3 and RAB10 are higher than 0.6, which revealed a well diagnostic performance of the six genes with HCC Figure S5A). These results revealed that the transcriptional levels of selected LD-localized proteins have prognostic value in HCC patients.

### NAFLD may contribute to the transcriptional different of PLIN3 in HCC patients

**Fig. 5:**
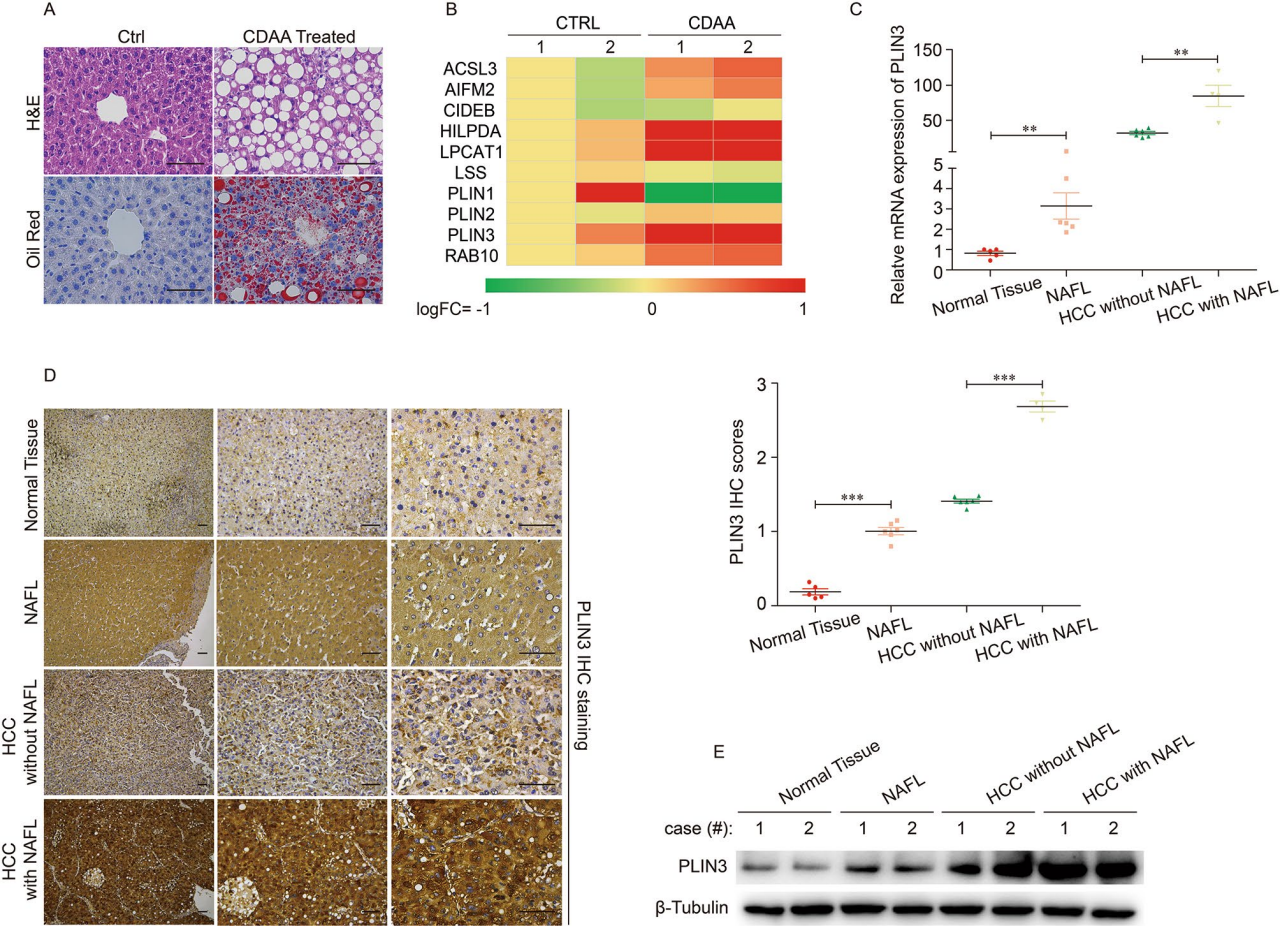
NAFLD may contribute to the transcriptional difference of PLIN3 in HCC patients. (**A**) The liver pathological feature and steatosis of mouse NASH model confirmed by H&E and Oil Red staining, respectively. (**B**) The transcriptome analysis revealed the expression levels of selected LDs localized proteins in the liver of mouse NASH model. (**C**) The mRNA expression of PLIN3 in the human liver specimen containing normal tissue (n = 5), NAFL (n = 6), HCC without NAFL (n = 6) and HCC with NAFL (n = 4). (**D**) The immunohistochemistry and immune scores for PLIN3 staining in human liver specimen containing normal tissue, NAFL, HCC without NAFL and HCC with NAFL. (**E**) Western blot analysis of PLIN3 protein level in in human liver specimen containing normal tissue, NAFL, HCC without NAFL and HCC with NAFL. **p < 0.01, ***p < 0.01

Considering the tight correlation of LDs accumulation and NAFLD, we hypothesized that the transcriptional change of the selected LDs localized proteins may attributed to the dysfunction of lipid metabolism in liver. Thus, we constructed mice model with LDs accumulation in the liver, and investigated the transcriptional change of selected LDs localized proteins. First, we constructed mice model with LDs accumulation in the liver by CDAA diet. H&E and Oil red staining confirmed the establishment of NASH model (Fig. 5A). Transcriptome analysis revealed the expression of *ACSL3*, *AIFM2*, *CIDEB*, *HILPDA*, *LPCAT1*, *LSS*, *PLIN1*, *PLIN2*, *PLIN3* and *RAB10* in the liver of control and CDAA treated mouse, and it was almost similar to the pattern in liver normal and tumor tissues we examined in online database and patients (Fig. 5B). Next, we took PLIN3 as an example to explore its expression in NAFL related HCC tissues. The mRNA and protein levels of PLIN3 in liver tissues, including normal tissue, NAFL, HCC without or with NAFL, were examined by qRT-PCR (Fig. 5C), immunohistochemistry (Fig. 5D), and western blot (Fig. 5E), which indicated that LDs accumulation in the liver could promote the transcription and protein expression of PLIN3. Moreover, compared with HCC tissues without NAFLD, the mRNA and protein levels of PLIN3 in HCC tissues with NAFLD were significantly increased. These results demonstrated that the hepatic steatosis partially contributed to the aberrant transcription of LDs localized proteins, which may be crucial for the higher mortality of HCC patients with hepatic steatosis.

### PLIN3 regulates the invasion and chemosensitivity of hepatoma cells

**Fig. 6:**
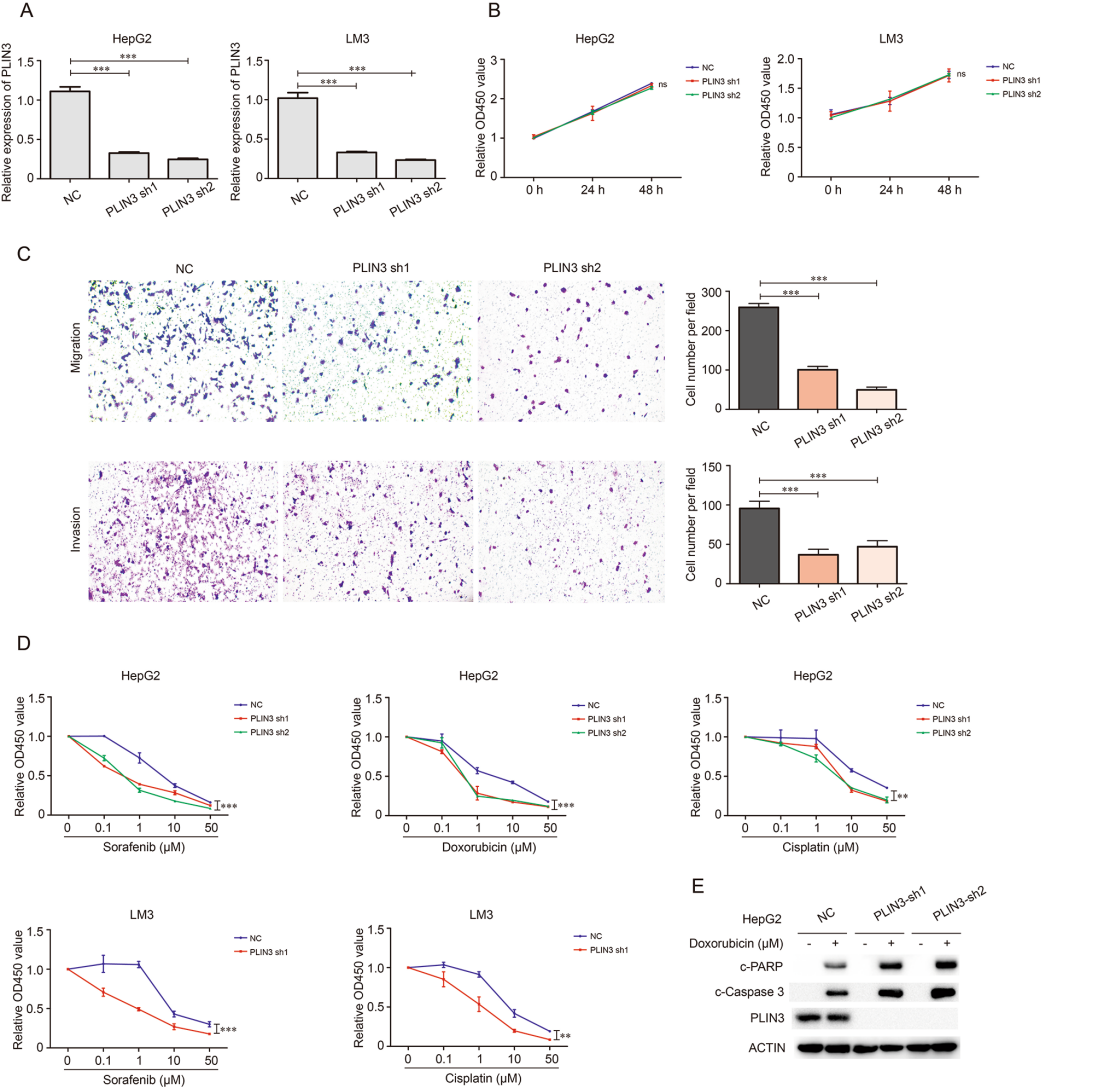
PLIN3 regulated the proliferation and chemosensitivity of hepatoma cells. (**A**) The knockdown of HepG2 and LM3 cells was constructed and determined by qRT-PCR. (**B**) The proliferation of HCC control and PLIN3 knockdown cells was evaluated by CCK-8 assay. (**C**) The migration and invasion of LM3 control and PLIN3 knockdown cells were identified by transwell assay. (**D**) The effects of sorafenib, doxorubicin and cisplatin on the proliferation of HCC control and knockdown cells were evaluated by CCK-8 assay. (**E**) The promotion of cytotoxicity induced by doxorubicin in PLIN3 knockdown hepatoma cells through apoptosis was identified by Western blot analysis. ns, no significance; **p < 0.01, ***p < 0.01

To further validate the function of PLIN3 in hepatoma cells, we first constructed PLIN3 knockdown cell lines, including HepG2 and LM3, by shRNA lentivirus, and the knockdown efficiency was confirmed by qRT-PCR (Fig. 6A). CCK-8 assay showed that PLIN3 had no effects on the proliferation of hepatoma cells (Fig. 6B). Besides, transwell assay revealed that the knockdown of PLIN3 remarkably impeded the migration and invasion of Hepatoma cells (Fig. 6C). Moreover, we explored if PLIN3 participated in the chemosensitivity of Hepatoma cells. The results showed that the knockdown of PLIN3 could enhance the cytotoxicity of sorafenib, doxorubicin and cisplatin in Hepatoma cells (Fig. 6D). Finally, we performed western blot to detect the apoptosis induced by these chemotherapeutic agents, and found that the cytotoxicity enhancement of PLIN3 knockdown in hepatoma cells may attributed to the increased apoptosis levels (Fig. 6E). These results demonstrated that PLIN3 strengthen the survival of hepatoma cells, and may contribute to HCC progression.

## Discussion

As highly dynamic organelles, LDs are actively participated in lipid storage and have an important role in lipid homeostasis, but their additional biological activities are rarely characterized. Recently, LDs have been considered as new hallmarks of cancer cells, their accumulation has been reported occurring in various cancer types and participating in mediating many aspects of cancer cells, including proliferation, invasion, metastasis, as well as chemotherapy resistance [12]. Furthermore, the increasing contents of LDs could offer energy source for proliferative cancer cells to satisfy their metabolic needs, and the storage of excessive FAs and cholesterol in LDs could also help them preventing lipotoxicity and ER stress. In addition, the increasing number of LDs is also a characteristic of hepatic steatosis, which could increase the risk for steatohepatitis, liver cirrhosis, and even hepatocellular carcinoma (HCC) [26]. Thus, investigating the regulation of LDs dynamic is a potential strategy of HCC treatment. While directly manipulating the LDs dynamic is difficult, targeting to the LDs localized proteins, which are the main regulators of LDs metabolism, is a more prospective way to investigate the LDs dynamic in HCC.

The main function and dynamic of LDs are carried out by LDs localized proteins. Although the LDs proteome analysis in mammalian cells has revealed that approximately 150 proteins associated with LDs, our literature research found that few of these proteins has been verified to localize on the surface of LDs by immunofluorescence, which is the most direct way to demonstrate the association between LDs and LDs localized proteins. The verified proteins localized on LDs are crucial for the origination, transportation, and fusion of LDs, and involved in multiple biological processes, such as lipid metabolism and signaling, redox metabolism, autophagy, gene transcription, ubiquitination, membrane trafficking and immunity [17, 27]. As core members of lipid metabolism and storage, LDs localized proteins also play an important role in liver steatosis, but there are few studies focusing on their specific function and mechanisms in the occurrence and development of HCC.

In current study, we first summarized the LDs localized proteins verified by immunofluorescence through literature research. Then, by transcriptional analysis, we found that 13 LDs localized proteins are differentially expressed in HCC, and have prognostic value for HCC patients. Among them, *ACSL3*, *AIFM2*, *HILPDA*, *HSD17B7*, *LPCAT1*, *LSS*, *PLIN3* and *RAB10* were upregulated and *CIDEB*, *G0S2*, *HSD17B13*, *PLIN1* and *PLIN2* were downregulated in HCC tissues compared to normal tissues based on GEPIA database. To verify these results, we examined their expression in tissue samples we collected and got the similar consequences. Next, we confirmed their protein expression by using the Human Protein Atlas database, and the results showed that the majority of protein levels corresponded to mRNA transcripts other than HSD17B7, but there were no immunohistochemistry results of G0S2 and PLIN2 identified in this database. Besides, the analysis of prognostic value further revealed the important roles of these factors in HCC.

Among the LDs localized factors we analyzed, there are three perilipins (PLINs), namely PLIN1, PLIN2 and PLIN3. PLINs are one of the most abundant LDs localized proteins, and comprise five members, including PLIN1, PLIN2, PLIN3, PLIN4 and PLIN5, which have been numbered in the order of their discovery [28]. PLIN2 and PLIN3 are ubiquitously expressed, while PLIN1, PLIN4 and PLIN5 have more limited tissue expression. PLINs can be used as a barrier for LDs decomposition, therefore help stabilizing triglycerides in LDs and promote their biosynthesis, thus promoting the growth of LDs. In addition, PLINs can also directly control how and when the cells and tissues to store, mobilize and use lipids, and they are the important regulators of lipid metabolism. Therefore, PLINs could play an important role in obesity, diabetes, liver disease and cancer caused by abnormal lipid metabolism.

In the liver, PLIN2 and PIN3 are two main functional LDs localized proteins, their expression levels on the LDs surface are dynamic in response to cellular metabolic needs, which can regulate the accessibility of enzymes involved in the lipid synthesis or lipolysis. PLIN2 would be degraded if displaced from the LDs, therefore it is a nonexchangeable protein [29]. However, PLIN3 is a highly exchangeable protein involved in various processes, such as lipid storage, lipid mobilization, and LDs biogenesis [30, 31]. In addition, PLIN3 is stable in the cytosol in quiescent stage and can be recruited to nascent LDs immediately when lipid storage is stimulated, and its knockdown could result in the inhibition of growth and maturation of LDs in cells even if stimulated by oleic acid [30]. In our informatics analysis based on public databases, PLIN3 was highly expressed in HCC tumor tissues, and positively correlated with the tumor grade and stage of HCC patients, while the expression pattern of PLIN2 was completely opposite. As for their prognostic values, the HCC patients with high expression of PLIN3 possessed poor prognosis, while the expression of PLIN2 was not related to the prognosis of HCC patients. It was indicated that PLIN3 was more positively correlated with the development of HCC. Thus, we took PLIN3 as a targeting protein to reveal the function of LDs localized proteins in the malignant progression of hepatoma cells.

The majority of current researches focused PLIN3 on its role in liver steatosis [32, 33], only a few studies paid attention to its function in cancers [34–36], and there has not a single study connecting PLIN3 with HCC so far. In our study, we confirmed that the protein levels of PLIN3 in HCC tissues is certainly associated with hepatic steatosis, and found that PLIN3 has the highest expression in HCC tissues accompanying with NAFLD. Knockdown of PLIN3 in hepatoma cells affected their migration and invasion, and it further had an influence on the cytotoxicity and apoptosis of sorafenib and doxorubicin on hepatoma cells, indicating that PLIN3 could affect the chemosensitivity of hepatoma cells. The mechanism of PLIN3 in sorafenib and doxorubicin induced the apoptosis of hepatoma cells may be attributed to autophagy. LDs have been recognized as the site for autophagosome biogenesis [37], and chaperone-mediated autophagy (CMA) is required to degrade PLIN3 before the selective sequestration of LDs by the autophagosome, which means that before autophagosome biogenesis associated and initiated by ATGs, PLIN3 would have to be removed from LDs surface [38], but the specific function of this mechanism on HCC development need to be further investigated. In our CDAA induced mouse NASH model, we found that the liver steatosis contributed to the expression changes of LDs localized proteins including PLIN3. Combine with the function of PLIN3 identified in hepatoma cells, we speculated that PLIN3 might be an important factor in the progression of NAFLD to HCC. However, this needs further intensive investigation and the exploration of the specific role of PLIN3 in the NAFLD-HCC pathogenesis would help to identify new targets for the diagnosis and prognosis of respective pathologies.

NAFLD is characterized by augmented accumulation of LDs, and the alteration in LDs metabolism has been proposed as a new hallmark of cancer [39]. Therefore, the changes in the expression of LDs localized proteins may be the accelerators in the progression of NAFLD to HCC. Moreover, the expression changes of these proteins may connect the hepatic steatosis with the malignance advancement of HCC. Thus, elucidating the mechanisms underlying this process is important for the development of new treatments and new therapeutic targets. Targeting to the LDs localized proteins could be the potential ways for the precision medicine of HCC patients with NAFLD.

## Electronic supplementary material

Below is the link to the electronic supplementary material.

Supplementary Material 1

## Data Availability

The original datasets of RNA sequence generated and analyzed during the current study have been uploaded on the GEO database (GSE223446), and can be downloaded from https://www.ncbi.nlm.nih.gov/geo/query/acc.cgi?acc=gse223446.
