# Supplementary material for: Transcriptional analysis of the expression and prognostic value of lipid droplet-localized proteins in hepatocellular carcinoma
Source: BMC Cancer. 2023 Jul 18;23:677. doi: 10.1186/s12885-023-10987-z (PMC10354995; doi:10.1186/s12885-023-10987-z)
Supplement: Supplementary file 1 — Supplementary Material 1 [file 12885_2023_10987_MOESM1_ESM.docx]

**Supplemental Information**

**Transcriptional analysis of the expression and prognostic value of lipid droplet-localized proteins in hepatocellular carcinoma**

**Yize Zhang, Xue Liang, Qinghai Lian, Liwen Liu, Baoyu Zhang, Zihui Dong, Kunpeng Liu**

**Contents of** **supplemental information**

**Supplemental figures S1-S5**

**Figure S1**


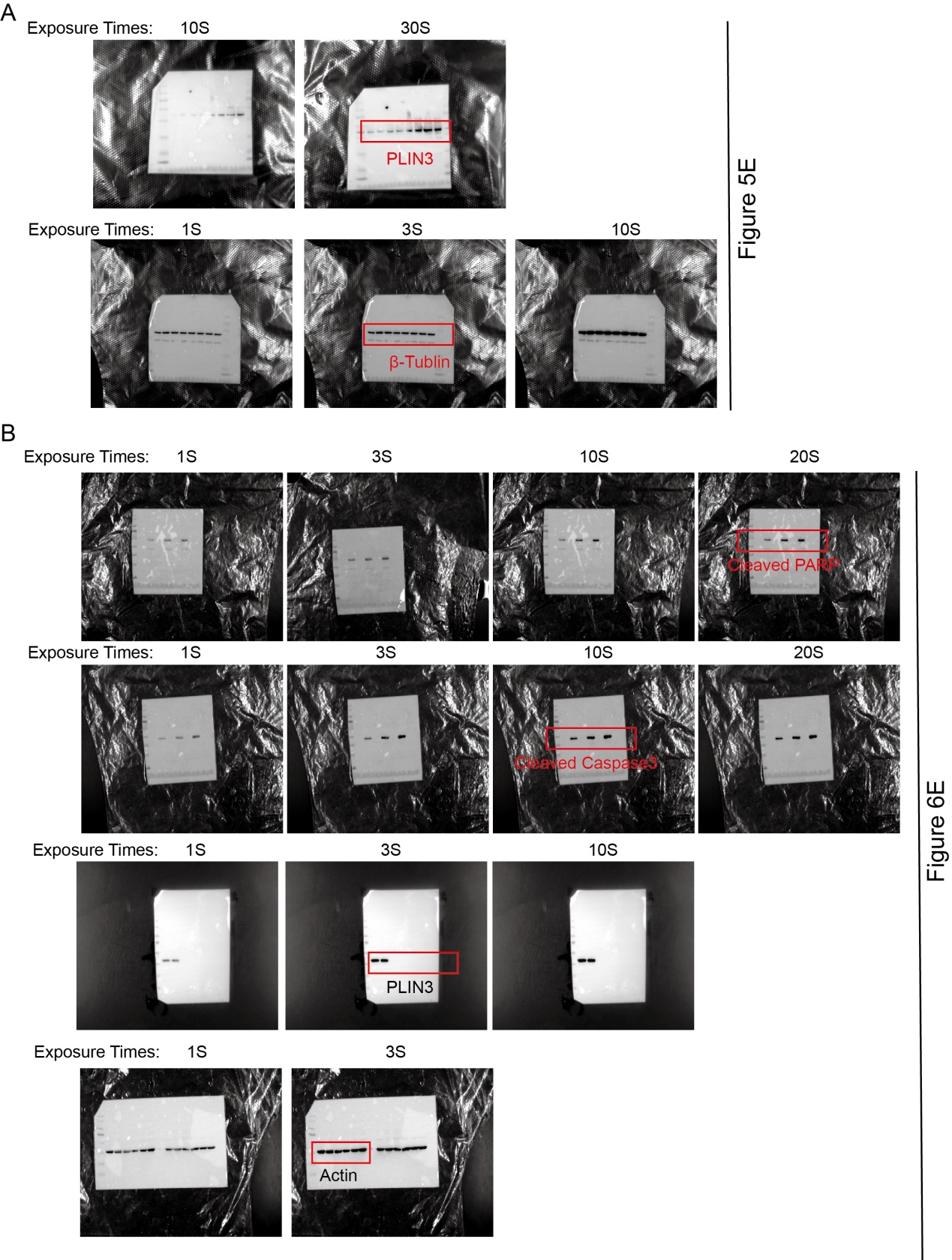


**Figure S1** (A) Exposure images of Figure 5E in manuscript. (B) Exposure images of Figure 6E in manuscript.

**Figure S2**


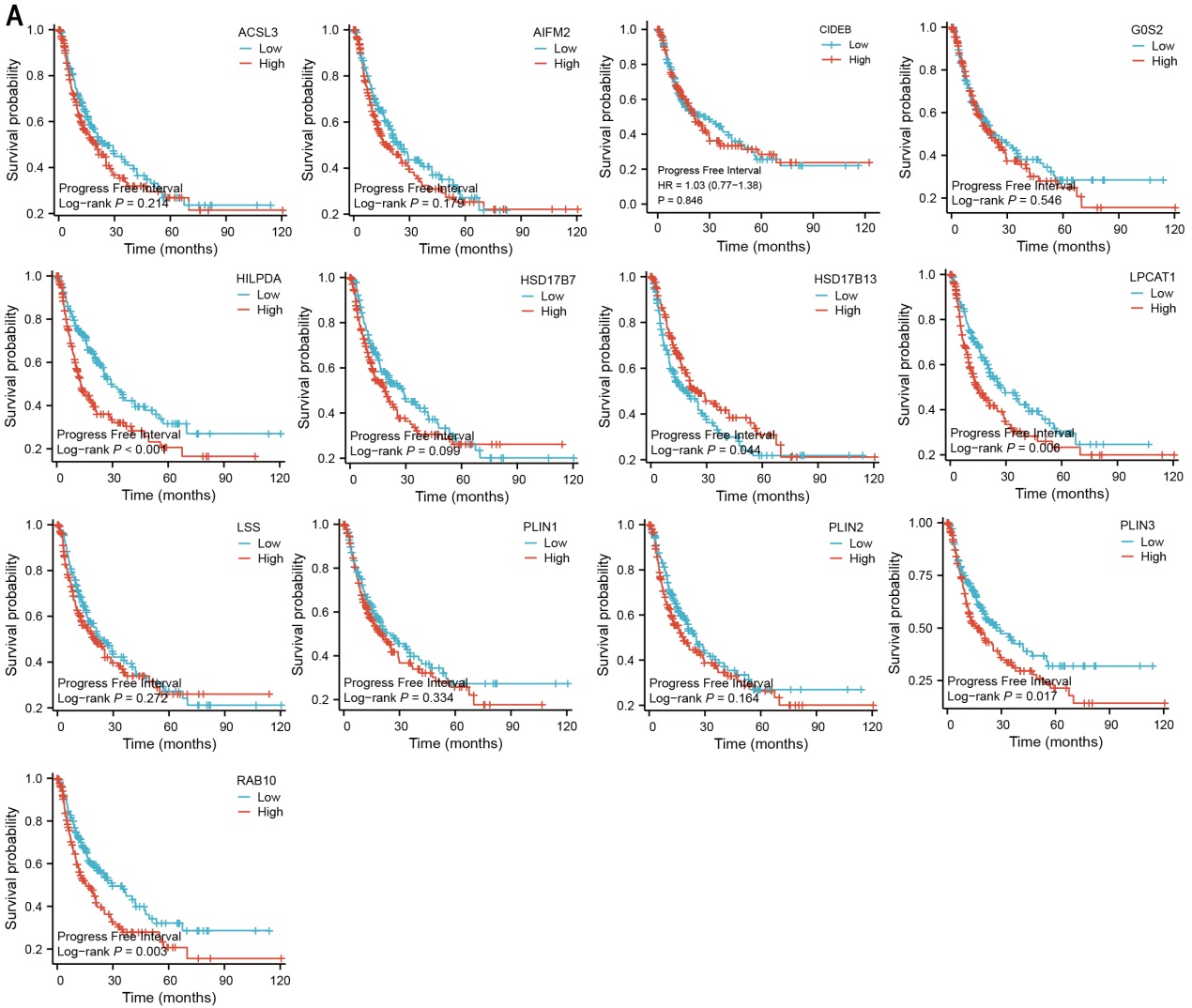


**Figure S2** (A) PFI analysis of selected proteins in HCC.

**Figure S3**


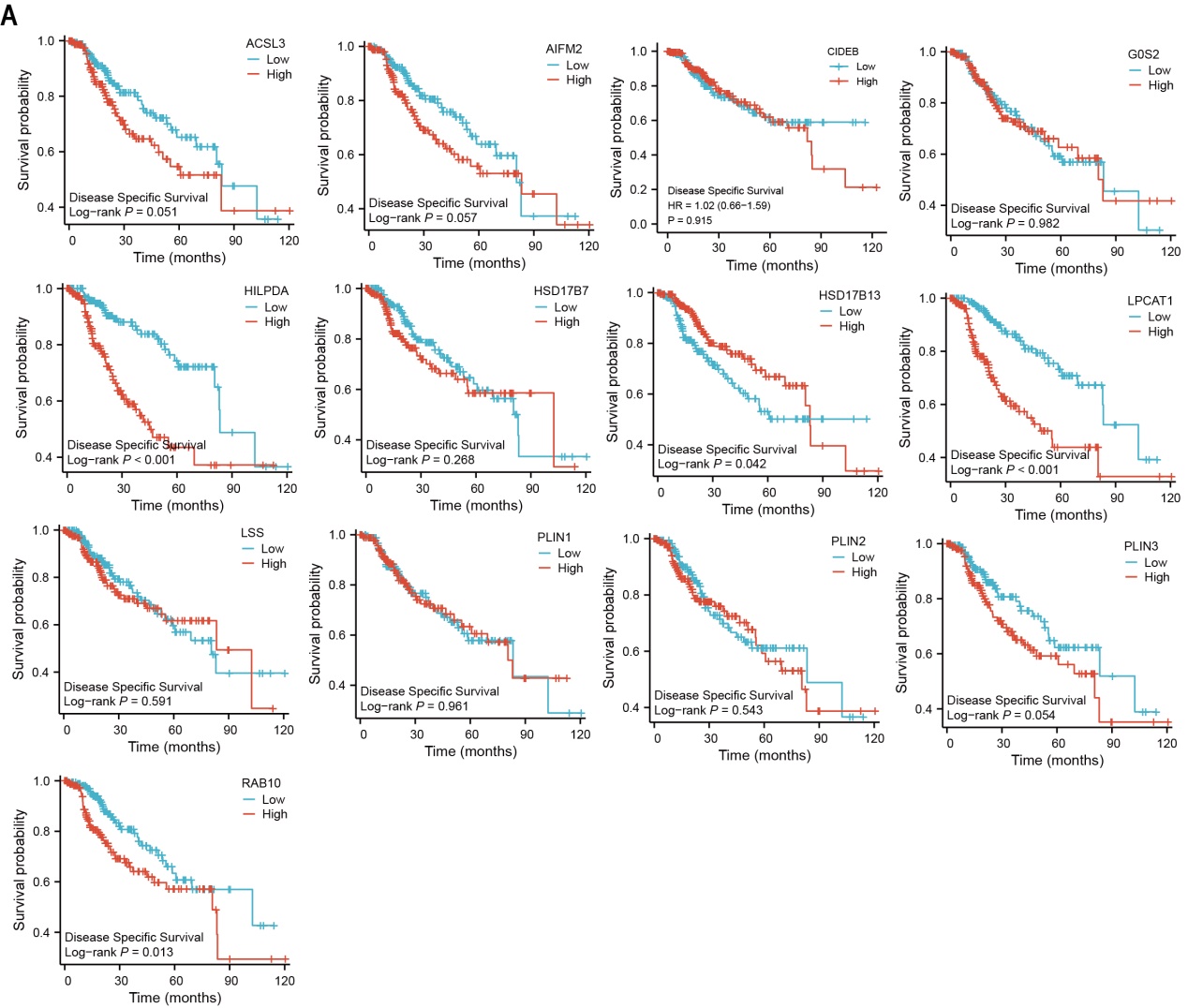


**Figure S3** (A) DSS analysis of selected proteins in HCC.

**Figure S4**


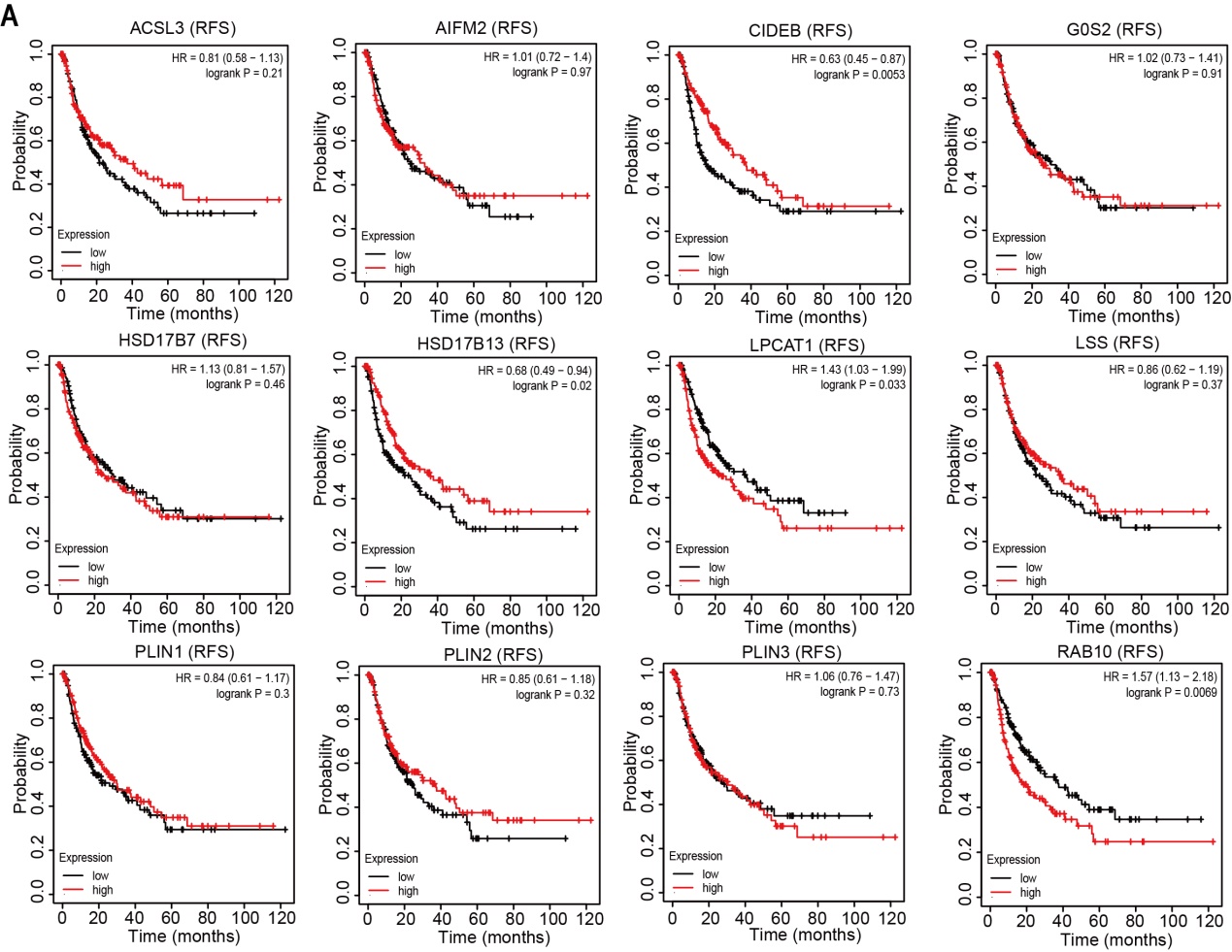


**Figure S4** (A) RFS analysis of selected proteins in HCC.

**Figure S5**


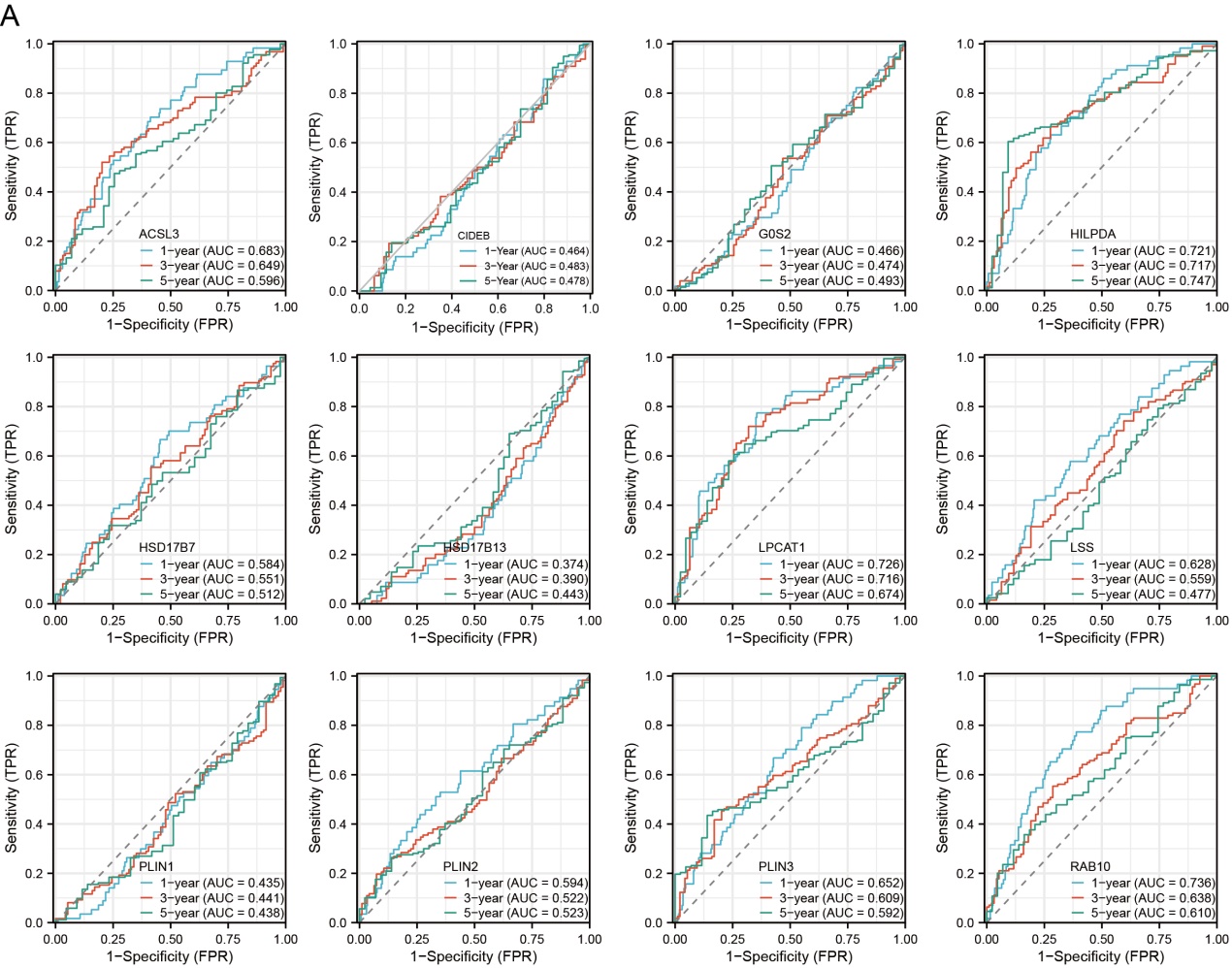


**Figure S5** (A) Time-dependent of ROC analysis of selected proteins in HCC.
